# Diagnostic Value of IgG and IgM Antibodies in Breastfeeding Mothers Infected With Cytomegalovirus (CMV)

**DOI:** 10.1155/sci5/8866962

**Published:** 2025-11-14

**Authors:** Faryal Saad, Sumaira Shams, Noor Ul Akbar, Sultan Ayaz, Naveed Ahmad, Mohammad Mahtab Alam, Muhammad Fayyaz Ur Rehman, Muhammad Safwan Akram

**Affiliations:** ^1^Department of Zoology, Abdul Wali Khan University Mardan, Mardan 23200, Khyber Pakhtunkhwa, Pakistan; ^2^Department of Zoology, Kohat University of Science and Technology, Kohat 26000, Khyber Pakhtunkhwa, Pakistan; ^3^Faculty of Chemical and Life Sciences, Abdul Wali Khan University Mardan, Mardan 23200, Khyber Pakhtunkhwa, Pakistan; ^4^Aquatic Diagnostic & Research Center Bahria University Karachi, Karachi 75260, Pakistan; ^5^Department of Basic Medical Sciences, College of Applied Medical Science, King Khalid University, Abha 61421, Saudi Arabia; ^6^Institute of Chemistry, University of Sargodha, Sargodha, Pakistan; ^7^School of Health and Life Sciences, Teesside University, Middlesbrough TS1 3BX, UK; ^8^National Horizons Centre, Teesside University, Darlington DL1 1HG, UK

**Keywords:** cytomegalovirus (CMV), ELISA, IgG, IgM, miscarriage

## Abstract

**Background:**

This study uses an ELISA-based cytomegalovirus (CMV) antigen estimation method to identify IgG and IgM antibodies in mothers' breast milk. In Pakistan, the prevalence of CMV is very high in the general population, especially in the province of Khyber Pakhtunkhwa (KPK), where it is at its worst due to poor healthcare practices, including poor sanitation, sterilization, suboptimal medicinal doses, and miscommunication between healthcare providers and patients, contributing to higher mortality rates. The high CMV prevalence has significant implication in infants including congenital infection and in mothers, health complications such as fatigue, flue, and jaundice. The study aims to determine the viral load of the CMV in breastfeeding women in KPK, Pakistan.

**Methods:**

Breast milk samples were collected in sterilized vacutainers from feeding mothers visiting hospitals in KPK. Each woman was asked about CMV infection through a background questionnaire. Milk samples were tested for CMV-specific immunoglobulin IgG and IgM antibodies.

**Results:**

A total of 406 breast milk samples from breastfeeding women in the research area were randomly analyzed. By using ELISA, 184 of them were found to have IgG positivity, and 49 had IgM positivity. These positive women were further segregated according to their education, age, socioeconomic status, place of residence, history of jaundice, parity, and miscarriage.

**Conclusions:**

It was discovered that nursing women face the same risk of infection, regardless of their age. The awareness of CMV appears to improve with education. However, the population's poor economic standing was a primary contributing factor to CMV infections. Additionally, in the research location, CMV was more common in countryside rural areas compared to metropolitan. ELISA was extremely sensitive for identifying antibody and antigen reactivity in individuals with recurrent and primary CMV infections.

## 1. Introduction

Human cytomegalovirus (CMV) is a species-specific DNA virus of the Herpesviridae family, prevalent worldwide, especially in lower socioeconomic countries [1]. It is the most common cause of mental retardation and hearing loss in fetus during pregnancy [2]. Breast milk is proposed as a vector in the transmission of HCMV in the first year of life [3]. Symptoms associated with CMV infection from breast milk, including neutropenia, lymphocytosis, thrombocytopenia, and hepatosplenomegaly, were reported first in 1983 [4, 5]. In 1988, a high rate of very low-birth-weight infants was reported with increased CMV infection symptoms. The most consistent diagnosis of viral load depends upon different factors, such as optimum sampling time with respect to the duration of the disease infectivity period, sampling material, and transfer of samples to the laboratory. Enzyme-linked immunosorbent assay (ELISA) is a common laboratory technique for the precise diagnosis and an antigen-based quantification of causative agents [6]. During screening, a high IgG titer was found when compared to IgM [7].

Preterm babies are physically immature and are vulnerable to CMV infection. They acquire the CMV virus postnatal through breast milk, showing the signs of infection. CMV-infected infants are generally asymptomatic for the infection but can shed the virus in urine and other body fluids [8, 9]. Zuhair and his colleagues conducted a systematic survey of available literature to estimate global burden of CMV. The CMV seroprevalence calculated by IgG antibodies was found to be 83% (95% UI: 78–88) in the general population, 86% (95% UI: 83–89) in women of childbearing age, and 86% (95% UI: 82–89) in donors of blood or organs. Among these three groups, the highest rate of prevalence was in the World Health Organization (WHO) Eastern Mediterranean region at 90% (95% UI: 85–94), and the lowest in the WHO European region at 66% (95% UI: 56–74). These estimates of CMV global distribution provide national and international models and show the need of developing a vaccine in near future [10, 11].

Pakistan is among the countries with one of the highest pregnancy-related death ratios, with overall maternal mortality rate of 260 per 100,000 and about 10% of the females of reproductive age die due to CMV infection. Moreover, Pakistan has an estimated neonatal and infant mortality rate of 85–299 per 100,000 annually, which accounts for 7% globally [12, 13]. CMV is a common viral infection, causing serious illness in healthy and especially in immunocompromised individuals showing no sign of infection at an early stage but later on causes severe health problems including hearing loss and neurological disorders. This creates a significant health burden globally. Henceforth, CMV has broad impact on individual health as well as global public health system. Human CMV can be transmitted to the fetus after delivery through the breast milk. In Pakistan, limited research is available on CMV prevalence in breastfeeding mothers.

In Pakistan, the high pervasiveness rate and endemicity of disease seem to be linked with socioeconomic status, residency, and climatic factors. Many communities in Pakistan have limited access to health facilities, poor hygiene, overcrowded residences along with low socioeconomic status, and limited awareness about this infection, which are the contributing factors. So these socioeconomic challenges highlight the importance of public health strategies to implement in deprived areas [9]. Henceforth, this study was proposed to test this hypothesis through ELISA for suspected CMV in breastfeeding mothers in different parts of Khyber Pakhtunkhwa (KPK), Pakistan.

## 2. Materials and Methods

### 2.1. Field of Study

**Figure 1:**
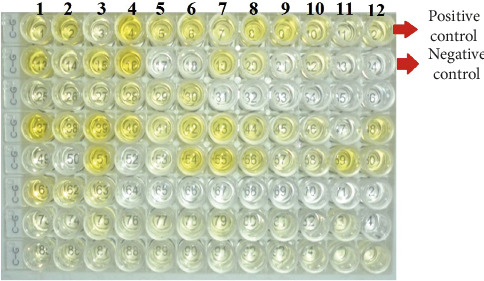
ELISA microplate showing positive cases for IgM antibodies with yellow color; positive control is in well no. 12, and negative control is in well no. 24 (both are marked).

One of Pakistan's four administrative provinces, KPK, is situated in the northwest and has a border with Afghanistan (Figure 1). The current research was performed in its different cities, namely Lakki Marwat, Bannu, Peshawar, and Swabi.

### 2.2. Selection of Samples

Breast milk samples were obtained from nursing women who visited KPK hospitals in antiseptic vacutainers (falcon tubes). Using a specially designed research questionnaire, each woman was asked about socioeconomic and health status. Identifying facts included age, education, family history, knowledge and awareness, and occupation. Women who were breastfeeding their young one were included in this study as a primary group to specifically investigate the CMV infection transmission dynamics, while women who were not breastfeeding were excluded from the study as they were unrelated to the key research question.

Milk samples were separated into cellular and aqueous components by centrifugation at 10,000 rpm to isolate CMV-specific IgM and IgG antibodies. Subsequently, these samples were delivered to the Zoology department of Abdul Wali Khan University Mardan (AWKUM) and stored at −20°C for future investigation. The Internal Review Board and the ethical committees of the individual hospitals approved the study's premise, questionnaires, and protocols of the study.

### 2.3. ELISA Test

Separate CMV-specific antibody IgM and IgG antibody tests were conducted on samples (milk) according to the manufacturer's instructions by utilizing an ELISA kit from Cenix Diagnostic GmbH (Dresden, Germany lot, M15DE). The samples' absorption wavelength during the ELISA analysis of positive cases was 450 nm. The 96-well ELISA microplate included negative and positive controls.

### 2.4. Interpretation of Results

The results were interpreted by microbial or microplate reader. We used the calorimetric method in which microwell reader read the optical density of the sample at 450 nm. The cut-off value was calculated as 2.1 of the negative control O.D. So, a sample was considered positive if its optical density (O.D.) was greater than 0.09 and negative if its O.D. was lower than 0.09.

### 2.5. Statistic Evaluation

Using Statistix Version 9 software, the results were statistically evaluated using the chi-square and one-way ANOVA test. Sensitivity was found as (true positive results/true positive + false negative results) × 100, and specificity was calculated as (true negative results/true negative + true positive results) × 100. Descriptive quantitative tests, such as percentages, assessed age and other clinical and demographic characteristics. SPSS version 16 was also used to calculate probability ratios.

## 3. Results

The study analyzed prevalence of CMV infection with regard to age, education, place of residence, employment, and relevant clinical characteristics. The results showed that CMV infection was significantly different in respective age groups, with high IgG antibodies prevalence in elderly groups. Education level influenced the infection rate; those who could not read were significantly affected in higher numbers and tested positive. It was also observed that place of residency of the people had close correlation with infection rate as rural residents presented more CMV prevalence as compared to urban residents (52.58% vs. 26.96% for IgG; 14.43% vs. 6.09% for IgM). Another critical finding of the current study was that unemployed were tested positive in higher numbers than employed. In clinical perspectives, jaundice, parity, and miscarriage history were shown to be associated with higher rates of CMV exposures.

### 3.1. Age-Related Prevalence

People in the study area were separated into three age groups (I–III) to examine the link between age and the prevalence of CMV infection (Table 1). In the age group I (12–23 years), 249 participants were tested, where 3.21% (*n* = 8) tested positive for IgM and 27.31% (*n* = 68) for IgG antibodies by ELISA (Tables 1 and 2).

Out of 109 people in age group II (24–35 years), 28.44% (*n* = 31) tested positive for IgM antibodies and 70.64% (*n* = 77) for IgG by the ELISA. Among women in age group III (36–47 years), 20.83% (*n* = 10) and 81.25% (*n* = 39) were positive for IgM and IgG immunoglobulin, respectively (Tables 1 and 2). The results indicate a clear trend of increasing IgG seropositivity with age.

### 3.2. Education-Related Prevalence

The effect of education on disease incidence was examined in the research area. Illiterate, Primary, Matric (O-levels), F.A/FSC (A-levels), Graduate, and Master were selected as six levels of education. The total number of illiterate people was 166 (40.88%), of whom 15.06% (*n* = 25) screened positive for IgM antibodies and 66.87% (*n* = 111) tested positive for IgG antibodies (Tables 3 and 4). The primary level of education also has a different relationship to the frequency of CMV; the ELISA results showed a prevalence of 43.78% (*n* = 43) for IgG and 11.22% (*n* = 11) for IgM. The prevalence of IgM antibodies was 9.37% (*n* = 3) for IgM, and for IgG, it was 21.87% (*n* = 27) in Matriculates. According to data from F.A/F.Sc, the CMV prevalence rate was 9.38% (*n* = 3) for IgM and 21.88% (*n* = 7) for IgG (Tables 3 and 4). Additionally, 8.69% (*n* = 2) and 13.04% (*n* = 3) of nursing mothers tested positive for the targeted antibodies (IgM and IgG) with odd values of less than 1, respectively.

### 3.3. Prevalence Based on Residence and Income

The vicinity of the residence to a major sewer, whether rural or urban, closely relates to the development of an infection, as in Pakistan, sanitary conditions vary widely between rich and poor areas. This study finds a close connection between poor living conditions, especially in rural areas lacking a sound sanitation system and hygienic environment, and an increased risk of CMV infection. Therefore, these factors are considered critical in spread of this infection. About 291 individuals (71.67%) in the researched region were inhabitants of the rural areas, where 14.43% (*n* = 42) and 52.58% (*n* = 153) tested positive for IgM and IgG antibodies, respectively; 115 individuals (28.32%) were inhabitants of the town, where 6.09% (*n* = 7) and 26.95% (*n* = 31) tested positive for IgM and IgG, respectively (Tables 5 and 6). According to the study results, CMV prevalence and personal finances were closely related. Mothers in the study provided a great example of this correlation. Financial impairments commonly lead to poor living standards, inadequate access to health facilities, lack of routine medical checkups, malnutrition, and unhygienic practices, which in turn increase the exposure to CMV infection. The economic conditions–based incidence of CMV included 286 (70.44%) unemployed mothers, of whom 43 (15.03%) were positive for IgM and 51.39% (*n* = 147) were IgG positive by ELISA. The prevalence rate for IgM antibodies was 5.00% (*n* = 6), and for IgG antibodies, it was 30.83% (*n* = 37) among working mothers (Tables 5 and 6).

### 3.4. Incidence-Relevant Clinical Characteristics (Miscarriage, Parity, and Jaundice)

The prevalence of CMV viral load in the studied group was examined through various clinical characteristics such as jaundice, parity, and a history of miscarriages. The chi-square test was used to examine the data statistically. Multiparous (who had previous children) and primiparous (experiencing first pregnancy and delivering first baby) mothers' illness prevalence rates were categorized into two. For IgM and IgG immunoglobulin separately, illness incidence was higher in multiparous individuals (13.97% and 52.57%, respectively) than in primiparous individuals (8.21% and 45.5%, respectively). Additionally, the odds ratio for IgM and IgG immunoglobulin was determined with a *p* value of ≤0.001 (Tables 7 and 8), while based on molecular detection, the study analyzed CMV epidemiology via polymerase chain reaction (PCR), showing an overall positivity rate of 18.97% among 406 cases. CMV was more prevalent in multiparous (21.32%) than primiparous women (14.18%), with significant associations found for jaundice history (*p* ≤ 0.001) and miscarriage (*p*=0.04) (Table S1).

Mothers' histories of jaundice were also examined because the infection incidence was correlated with jaundice, which was present in 77.5% (*n* = 38) of IgM-positive cases and 65.30% (*n* = 32) of IgG-positive cases. For both immunoglobulins, the record of abortion was also examined, and it was discovered to be higher at 53.02% (*n* = 158) for IgG-positive and 14.09% (*n* = 42) for IgM-positive groups, respectively (Tables 7 and 8). An odds ratio greater than 1 was discovered with a *p* ≤ 0.001 significance threshold, as demonstrated in Table 8.

**Figure 2:**
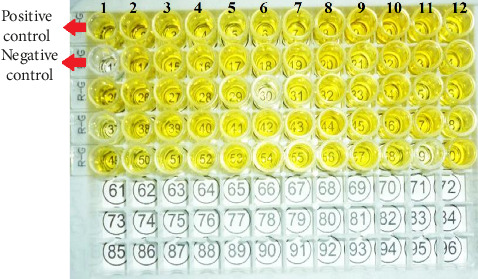
ELISA microplate showing positive cases for IgG antibodies; positive control is in well no. 1, and negative control is in well no. 13 (both are marked).

The ELISA microplate has 96 wells for both IgG and IgM antibodies, which include negative and positive control samples; in Figures 1 and 2, the yellow color denotes positive samples (Figures 1 and 2).

## 4. Discussion

This study focused on the seroprevalence of CMV infection in breastfeeding mothers. Questionnaires were distributed to all 406 breastfeeding mothers irrespective of their health conditions. In addition, some mothers were also suffering from lethal infections such as HIV, TB, etc., which made them susceptible to CMV infection being immunocompromised. The co-occurrence of these infections create vulnerable situation for CMV outcomes to become even worse, for instance, chronic T.B produces several inflammatory responses resulting in severe clinical manifestations. Moreover, CMV infection accelerates HIV cycle progression, which in turn creates complications in the medical treatment. Therefore, examining interactions between coexisting infections and CMV provides a valuable scenario with respect to CMV transmission. Breastfeeding mothers are very prone to CMV infection because of their close contact with baby's bodily secretions such as urine, saliva, and mother's milk itself, which are the common carrier for CMV transmission. Particularly, when mother had a previous history of CMV infection, it is possible to have reactivation creating mild to severe health risks. During breastfeeding, several immunological and hormonal changes occur, which in turn increase the susceptibility of infection. Breast milk is a natural immune potentiator and passive immunity carrier. Infected breastfeeding mothers create risk to the infants, especially for immunocompromised or premature infants. The preterm infants are more susceptible to CMV infection because of their underdeveloped immune system.

Mothers who had a child with stillbirth or born with jaundice were also interviewed. In the present study, we found a close relation between education level and prevalence of CMV infection. The study of Ibrahim et al. and Mujtaba et al. investigated CMV and its association with sociodemographic factors in the adult population of Pakistan, which showed that disease prevalence decreases as a certain population's education level increases. They provided a baseline prevalence rate of CMV infection and explained sociodemographic influences on CMV infection risk specific to the people of Pakistan [13, 14]. This can be attributed to the fact that with increased education levels, awareness among the population regarding particular infections increase, leading to overall increase in hygiene. In Pakistan's context, increased level of education directly relates to increased household income.

ELISA has been shown to be a reliable detection method, and this study found it to have a good sensitivity for detecting CMV infection. However, our results contradict previous observations [6] that noted limitations in serological assays like ELISA. For example, they described that in immunocompromised individuals, due to the decreased number of antibodies, the actual results are doubtful [15]. However, we found limit of detection to be quite sensitive and fit for purpose. Based on assay types and population demographics, ELISA usually displays performance statistics in terms of sensitivity from 85% to 95% and specificity from 90% to 98%. In contrast, PCR exceeds ELISA in sensitivity levels, because it detects viral DNA to confirm active CMV infections. PCR sensitivity reaches 95%–100%, and its specificity reaches 99% for detecting congenital CMV infections and active infections. Nevertheless, the ELISA continues to offer value in epidemiological research because it identifies individuals who have built immunity against CMV. Therefore, it stands as an ideal tool for population-level research; however, doctors use PCR to detect current CMV infections because of increased diagnostic accuracy [16]. In the present research, during active CMV testing, IgM demonstrated a sensitivity level of 63.6%, which resulted in false negatives for 36.4% of cases while maintaining 100% accuracy for all negative results (no false positives). By comparing to the PCR results (unpublished), the current ELISA has proven to be a reliable detection method, and this study found it to have moderate sensitivity (63.6%) and high specificity (100%) for detecting CMV infection in breast milk [17].

As described earlier, in the present study, we showed that the education level highly influenced awareness of CMV infection. Better hygienic practices are associated with higher education level, which can minimize the risk of CMV infection. Educated individuals have better understanding and awareness about viral transmissions. They usually practice standard protocols of hygiene well including regular hand washing and safe handling of urine and saliva. This reinforced preventive behavior lowers the risk of CMV infection inside populations. The prevalence of CMV antibodies showed an inverse and statistically significant relationship with level of education when used to measure previous infection (IgG antibodies; *χ*^2^ = 72.1, *p* ≤ 0.001). Conversely, recent infection (IgM antibodies) did not have a significant relationship with the level of education (*χ*^2^ = 3.0, *p*=0.70). Although the highest rate of IgM was recorded among the illiterate group (15.1), the differences among educational groups were not statistically significant. This indicates that the risk of a recent CMV infection is sensitive to specific, short-term exposures (e.g., contact with young children) rather than to broad socioeconomic status as reflected in educational attainment.

In addition, we found that individuals' economic status also significantly impacts CMV prevalence. We divided economic status into two (employed and unemployed) categories, in which women at low economic levels were at higher risk of infection with no monthly or daily income on regular basis than those at the middle/high economic level with regular monthly income for daily life expenses, as shown in Tables 3 and 4. Most members of Pakistan's population remain in the “low economic status” bracket according to the lower-middle-income poverty threshold of daily earnings of $3.2 which amounts to monthly income of $96. The defined threshold operates as a relevant indicator of “low income” because it matches data showing that 18.7%–25.3% of Pakistan's population lived below $3.2 per day poverty line in 2022. The economic challenges and severe floods throughout 2022 made conditions worse for low-income households, thus creating circumstances that fostered increased CMV transmission through overcrowding together with poor sanitation and restricted healthcare access. Studies of CMV risks in low-income populations should incorporate environmental and economic factors since the research confirms their importance [18].

In the present research, mothers with no regular income (unemployed, 190 out of 286) had low living standard and had high prevalence of CMV infection, while women with middle and high regular income (employed, 43 out of 120) had high and moderate living standards and had financial stability. The economic stability possibly reduces the exposure of CMV infection, by providing standard life style. Individuals of low-income and crowded families also had a high risk of transmission of CMV infection in the study area. In rural areas, nonavailability of infrastructure and people being mostly engaged in labor and agriculture place individuals at a higher risk of infection due to low personal status, low economy, poor hygienic conditions, and crowded situations. Most of the time, poor economic conditions are also associated with poor education, thus further increasing the risks of infection.

Comparatively, seroprevalence of CMV infection in rural areas was higher than in urban areas (more developed infrastructure, nonagricultural, and more healthcare and educational institutions), as shown in Tables 5 and 6. The high prevalence rate of CMV infection in rural areas can also be attributed to unhygienic environmental conditions at home. Many women lacked awareness of hygiene practices, such as hand washing after changing diapers, wiping the nose of the baby, and handling the baby's urine or stool. The lack of health facilities in rural areas and the inability to travel to big hospitals due to poor economic conditions increase the risks of infections. These findings that socioeconomic conditions are the main contributing factors to the CMV infection agree with previous observations [19], where it was found that abortion rate is directly related to CMV infection, which is also supported by our results. However, our results support the use of serological assay compared to PCR because of its cost-effectiveness and ease of use [7, 20].

The age of the study group was divided into three groups (I–III), where analysis of different age groups revealed that CMV infection is relatively constant (Group I = 68, Group II = 77, Group III = 39) in all three age groups of mothers. Conversely, the age-related stability in CMV prevalence need careful interpretation and sample sizes in every group because every single group members may have different life style and diverse immune responses that possibly will impact the findings of the study. For more accurate and precise observation, further studies with large sample size and controlled variables are necessary [20].

To see the impact of education on CMV prevalence, the education parameter was divided into six categories. The CMV infection prevalence rate was found to be inversely proportional with education. The women with higher education levels (graduates and masters) were less affected compared to the illiterate group of women. In the current study, we observed women with jaundice history, fever, and illness, and women with high parity were at a high risk of active CMV infection. We also observed women with jaundice history had miscarriages about one in four times. High parity factor increased the susceptibility to acquisition of maternal CMV infection, perhaps through direct contact with contagious secretions from their children or poor hygiene practices by these women. This study suggest that mother with more children are prone to CMV infection because of limited time and resources for precautionary healthcare practices. Due to large number of children, resources per family member might be fewer, which impaired their capacity to maintain a good hygienic environment. High parity also has emotional and physical demands, which in turn decline the mother's immune system, making them vulnerable to CMV infection. However, given the numerous causes of fever and jaundice, these represent a nonspecific indicator of maternal CMV infection [21, 22]. So far, most of the studies have been carried out and focused on finding an association of residency with CMV prevalence. Mothers with residences in rural areas have more infectivity than those with urban residences. HCMV's high prevalence rate may be due to poor living standards, unhygienic practices, and poor socioeconomic status [23, 24]. The utilization of the above-described ELISA kit for the analysis of breast milk represents an innovative approach, as prior investigations have predominantly focused on its application in serum analysis. Although less prevalent, there are instances of immunoassays being applied in the analysis of breast milk. Notably, Gang et al. utilized an ELISA technique to examine CMV antibodies in breast milk and sera, highlighting the viability of immunoassays for the evaluation of HCMV-specific antibodies in the breast milk [7].

The detection of 12.06% IgM antibodies, in contrast to IgG 45.32% in the present study, may point to an active CMV infection and might be startling indicators in these women, as previous studies reflect. The transfer of antibodies from blood to breast milk involves passive diffusion and active transport mechanisms, influenced by factors such as antibody type, size, charge, and receptor presence on mammary cells. While IgM antibodies are typically too large to cross certain barriers like the placenta, they are commonly found in breast milk along with IgA and IgG. These antibodies help provide immunity to nursing infants, with concentrations varying based on maternal health, immunization status, and lactation stage. In response to acute infections, IgM antibodies are initially released into the milk, followed by longer-lasting IgG antibodies, reflecting the mother's immune response status. Detection of IgG or IgM antibodies in breast milk indicates the mother's immune status [24, 25]. The research pertaining to IgM in breast milk is rather limited given its lower concentration as compared to IgA. Its half-life determination is quite complex as it is affected by multiple factors and the majority of existing literature mainly concentrates on secretory IgA and IgG. In this study, antibody levels in breast milk were not normalized to total protein concentration. We acknowledge that interindividual variability in milk composition, particularly total protein content, may influence the absolute concentrations of HCMV-specific IgG and IgM measured by ELISA. Although this introduces a potential source of variability, we minimized its impact by employing standardized sample volumes and consistent assay protocols across all specimens, thereby ensuring internal consistency within the dataset. Importantly, our analyses focused not only on absolute concentrations but also on the presence or absence of antibody reactivity and relative comparisons between groups. The consistency of these trends across multiple samples supports the robustness of our findings, even in the absence of protein normalization. Nonetheless, we recognize this as a methodological limitation. Future studies should incorporate normalization to total protein content, using assays such as BCA or Bradford, to enhance the accuracy and comparability of immunoglobulin quantification in breast milk. From the above study, it was also clear that IgG has a higher avidity of infection than IgM. Also, rural areas were at the peak position of infection compared to the study area's urban population. Education and economic level also significantly differed between the categories, as shown in the tables.

## 5. Conclusions

The frequency of CMV infection in breastfeeding mothers varies based on age, education, income, and location. Education and economic status influence CMV awareness and prevalence. CMV is more common in rural areas due to limited health facilities and awareness. Lower socioeconomic status and regional citizenship also affect infection rates. The study group has a lower prevalence due to cultural factors, such as behaviors, community interactions, social structure, and standards about hygiene. Cultural norms having limited interaction with non–family members and avoiding close proximity lowers the risk of infection. Improving diagnosis and awareness can reduce maternal and neonatal CMV transmission, positively impacting childhood health and mortality. There is an urgent need for routine screening to assess accurate burden of CMV infections. Furthermore, there is need to explore with a large sample size in different localities all over Pakistan. More research is necessary to facilitate potential CMV prevention and initiation of effective vaccination program against CMV infection.

## Figures and Tables

**Table 1:** Prevalence of CMV based on age diagnosed through ELISA for IgM antibodies.

Age (in years)	Total (*n*)	IgM antibodies				Odds ratio	*p* value
		Positive	%	Negative	%		
12–23	249	8	3.21	241	96.79	0.03	*p* ≥ 0.05
24–35	109	31	28.44	78	71.56	0.40	
36–47	48	10	20.83	38	79.17	0.26	

**Table 2:** Prevalence of CMV based on age diagnosed through ELISA for IgG antibodies.

Age (in years)	Total (*n*)	IgG antibodies				Odds ratio	*p* value
		Positive	Percent	Negative	Percent		
12–23	249	68	27.31	181	72.69	0.38	*p* ≥ 0.05
24–35	109	77	70.64	32	29.36	2.41	
36–47	48	39	81.25	9	18.75	4.33	

**Table 3:** Prevalence of CMV based on education diagnosed through IgG antibodies.

Educational level	Total (*n*)	IgG antibodies				Odds ratio	*p* value
		Positive	Percent	Negative	Percent		
Illiterate	166	111	66.87	55	33.13	2.02	*p* ≤ 0.001
Primary	98	43	43.88	55	56.12	0.78	
Matric	72	19	26.39	53	73.61	0.36	
F.A/F.Sc	32	7	21.88	25	78.13	0.28	
Graduate	23	3	13.04	20	86.96	0.15	
Masters	15	1	6.67	14	93.33	0.07	

**Table 4:** Prevalence of CMV based on education diagnosed through IgM antibodies.

Educational level	Total (*n*)	IgM antibodies				Odds ratio	*p* value
		Positive	Percent	Negative	Percent		
Illiterate	166	25	15.06	141	84.94	0.18	*p* ≥ 0.05
Primary	98	11	11.22	87	88.78	0.13	
Matric	72	7	9.72	65	90.28	0.11	
F.A/F.Sc	32	3	9.38	29	90.63	0.10	
Graduate	23	2	8.70	21	91.30	0.10	
Masters	15	1	6.67	14	93.33	0.07	

**Table 5:** Prevalence of CMV based on residential area and economic status diagnosed through IgG antibodies.

**IgG antibodies**							
	**Total (*n*)**	**Positive**	**Percent**	**Negative**	**Percent**	**Odds ratio**	**p** **value**

Residence							
Rural	291	153	52.58	138	47.42	1.11	*p* ≤ 0.001
Urban	115	31	26.96	84	73.04	0.37	
Occupation							
Unemployed	286	147	51.40	139	48.60	1.06	*p* ≤ 0.001
Employed	120	37	30.83	83	69.17	0.45	

**Table 6:** Prevalence of CMV based on residential area and economic status diagnosed through IgM antibodies.

**IgM antibodies**							
	**Total (*n*)**	**Positive**	**Percent**	**Negative**	**Percent**	**Odds ratio**	**p** **value**

Residence							
Rural	291	42	14.43	249	85.57	0.17	*p* ≤ 0.001
Urban	115	7	6.09	108	93.91	0.06	
Occupation							
Unemployed	286	43	15.03	243	84.97	0.18	*p* ≤ 0.001
Employed	120	6	5.00	114	95.00	0.05	

**Table 7:** Prevalence of CMV based on parity, history of jaundice, and miscarriage diagnosed through IgG antibodies.

**IgG antibodies**							
	**Total (*n*)**	**Positive**	**Percent**	**Negative**	**Percent**	**Odds ratio**	**p** **value**

Parity							
Primiparous	134	61	45.5	93	69.40	0.44	*p* ≤ 0.001
Multiparous	272	143	52.57	129	47.43	1.11	
History of jaundice							
Yes	49	32	65.31	17	34.69	1.88	*p* ≤ 0.001
No	357	152	42.58	205	57.42	0.74	
History of miscarriage							
None	108	26	24.07	82	75.93	0.32	*p* ≤ 0.001
1 to 4	298	158	53.02	140	46.98	1.13	

**Table 8:** Prevalence of CMV based on parity, history of jaundice, and miscarriage diagnosed through IgM antibodies.

**IgM antibodies**							
	**Total (*n*)**	**Positive**	**Percent**	**Negative**	**Percent**	**Odds ratio**	**p** **value**

Parity							
Primiparous	134	11	8.21	123	91.79	0.09	*p* ≤ 0.001
Multiparous	272	38	13.97	234	86.03	0.16	
History of jaundice							
Yes	49	38	77.55	11	22.45	3.45	*p* ≤ 0.001
No	357	11	3.08	346	96.92	0.03	
History of miscarriage							
None	108	7	6.48	101	93.52	0.07	*p* ≤ 0.001
1 to 4	298	42	14.09	256	85.91	0.16	

## Data Availability

Data are available upon request.

## Supporting Information

The data presented in ST1 is unpublished and is shared here for the purpose of demonstrating the sensitivity and specificity of diagnostic methods. The findings highlight key associations between CMV prevalence and clinical factors, reinforcing the need for targeted screening and preventive measures in affected populations.
