# Supplementary material for: Diagnostic Value of IgG and IgM Antibodies in Breastfeeding Mothers Infected With Cytomegalovirus (CMV)
Source: Scientifica (Cairo). 2025 Nov 14;2025:8866962. doi: 10.1155/sci5/8866962 (PMC12638136; doi:10.1155/sci5/8866962)
Supplement: Supporting Information — Additional supporting information can be found online in the Supporting Information section. [file 8866962.f1.docx]

**Table S1:** Epidemiology of CMV by PCR in relation to clinical features

| PCR | | | | | | |
| --- | --- | --- | --- | --- | --- | --- |
| **Parameters** | | **No Examined** | **%** | **Positive** | **Positive (%)** | **P value** |
| Parity | Primiparous | 134 | 33.00 | 19 | 14.18 | P ≥ 0.05 |
|  | Multiparous | 272 | 67.00 | 58 | 21.32 |  |
|  | Total | 406 |  | 77 | 18.97 |  |
| History of jaundice | Yes | 49 | 12.07 | 22 | 44.90 | P ≤ 0.001 |
|  | No | 357 | 87.93 | 55 | 15.41 |  |
|  | Total | 406 |  | 77 | 18.97 |  |
| History of miscarriage | None | 108 | 26.60 | 9 | 8.33 | P ≤ 0.05 |
|  | 1 to 4 | 298 | 73.40 | 68 | 22.82 |  |
|  | Total | 406 |  | 77 | 18.97 |  |

Note: A P value of less than 0.05 indicates statistical significance
